# Edible insects as functional foods: bioactive compounds, health benefits, safety concerns, allergenicity, and regulatory considerations

**DOI:** 10.3389/fnut.2025.1571084

**Published:** 2025-03-31

**Authors:** Fernando E. Alejandro Ruiz, Julio F. Ortega Jácome, Eduardo Tejera, José M. Alvarez-Suarez

**Affiliations:** ^1^Laboratorio de Investigación en Ingeniería en Alimentos (LabInAli), Departamento de Ingeniería en Alimentos, Colegio de Ciencias e Ingenierías, Universidad San Francisco de Quito USFQ, Quito, Ecuador; ^2^Laboratorio de Bioexploración, Colegio de Ciencias Biológicas y Ambientales, Universidad San Francisco de Quito USFQ, Quito, Ecuador; ^3^Grupo de Bioquimioinformática, Facultad de Ingeniería y Ciencias Aplicadas, Universidad de Las Américas (UDLA), Quito, Ecuador

**Keywords:** edible insects, bioactive compounds, food safety, allergens, functional foods

## Abstract

The growing demand for sustainable and nutrient-rich food sources has positioned edible insects as a viable alternative to traditional animal-based proteins. This review explores the bioactive properties and food safety considerations of edible insects, emphasizing their potential health benefits and the challenges associated with their widespread consumption. Research has identified bioactive compounds in insects with antioxidant, antimicrobial, immunomodulatory, cardioprotective, and digestive health-promoting properties, highlighting their potential as functional foods for preventing or managing chronic diseases such as cardiovascular conditions and inflammatory disorders. Additionally, this review examines findings related to contaminants in edible insects, including heavy metals, microbial pathogens, and allergens, which could pose health risks. Certain insect species have shown accumulation of heavy metals, such as cadmium and lead, depending on their diet and environment. Moreover, microbial contamination, including bacteria, fungi, and parasites, can occur if farming and processing conditions are not properly controlled. Furthermore, insect proteins exhibit cross-reactivity with allergens found in crustaceans and dust mites, raising concerns for individuals with food allergies. For edible insects to be successfully integrated into global food systems, further technological advancements, regulatory oversight, and consumer acceptance strategies must be implemented. Addressing these challenges will enable edible insects to become a key component of sustainable food systems, contributing to global nutrition, environmental sustainability, and human health.

## 1 Introduction

The rising global attention to edible insects as a novel and eco-friendly food option highlights an increasing awareness of their potential to address two critical challenges of our time: guaranteeing a stable food supply and supporting ecological sustainability (1). As estimates predict a global population exceeding 9 billion by 2050, the urgency for nutrient-dense and environmentally responsible food sources continues to grow (2). Conventional animal agriculture, while a major source of protein, imposes significant environmental burdens, including deforestation, biodiversity loss, greenhouse gas emissions, and extensive water usage. These circumstances have prompted researchers and decision-makers to investigate new protein alternatives capable of fulfilling the nutritional requirements of an expanding population without intensifying environmental harm.

Given their high nutritional value and low environmental impact, edible insects emerge as a promising alternative to tackle the global protein deficit and promote more sustainable food production systems (1). As well as being a source of high-quality protein, edible insects provide essential fats, vitamins, and minerals, making them a nutrient-dense food item that can compete with traditional livestock in terms of nutritional output (3). Additionally, insects contain bioactive compounds such as chitin, polyunsaturated fatty acids (PUFAs), peptides, and antioxidants, which contribute to their functional properties (3). Research indicates that these substances possess antioxidant, antimicrobial, and immunomodulatory effects, implying that edible insects could provide health advantages beyond essential nutrition. Bioactive components in insects could play a part in preventing or managing chronic diseases, e.g., cardiovascular disease, diabetes, and inflammatory conditions, positioning them as potential functional foods or nutraceuticals (4). The importance of edible insects in the global food system is further underscored by their role in traditional diets in various regions across the globe. In many areas of Africa, Asia, and Latin America, insects have been consumed for centuries, not only for their nutritional value (5) but also for their medicinal properties (6). In these cultures, insects have been employed in entomotherapy to treat a wide range of issues, including inflammatory disorders, infections, and pain. The therapeutic use of insects, while historically empirical, has prompted recent scientific exploration into their potential health benefits (6). Studies on the bioactivity of edible insects have begun to elucidate the mechanisms behind their medicinal uses, revealing their potential as sources of useful bioactive compounds that could be developed for pharmaceutical or nutraceutical applications (4). As research in this area expands, the role played by edible insects in both nutrition and health is likely to gain greater recognition and acceptance However, despite the nutritional and functional advantages of edible insects, their widespread adoption as a mainstream food source faces several challenges. One of the most critical issues is food safety. Insect farming and processing can introduce risks of microbial contamination, including bacteria, viruses, and parasites, which must be carefully managed to ensure consumer safety (7). Regulatory frameworks created by entities such as the European Food Safety Authority (EFSA) are crucial for setting standards to mitigate these risks and ensure that insects are produced and processed under conditions that meet food safety requirements (8). In addition to microbial contamination, the presence of antinutrients in insects may interfere with nutrient absorption, necessitating further research into how these compounds impact the bioavailability of key nutrients (9). Recognizing the possible effects of antinutrients is essential for maximizing the nutritional benefits of edible insects.

Another significant concern is allergenicity. Some insect proteins are structurally similar to allergens found in shellfish and crustaceans, raising the possibility of allergic reactions in sensitive individuals (10). This issue is particularly relevant as edible insects become more integrated into global food markets, where individuals with seafood allergies may find themselves at risk of cross-reactivity. Identifying and characterizing insect allergens, as well as developing appropriate labeling and allergen management strategies, will be essential for ensuring the safe consumption of insect-based products. Additionally, public awareness campaigns may be needed to educate consumers about potential allergens associated with insect consumption. Beyond safety concerns, the success of integrating edible insects into the global food supply will depend on the development of comprehensive regulations and standardized farming practices. As seen with the adoption of edible insects in Europe, regulation authorities such as the EFSA play a crucial role in shaping the market for insect-based foods by providing guidelines on their safe use and ensuring that consumers can trust the products available to them. Although consuming edible insects is a long-standing practice in various regions worldwide, their incorporation into everyday diets in areas like Europe and North America marks a substantial change in how we perceive food production and consumption. As the research and regulatory landscape continues to evolve, edible insects could become a key component of sustainable food systems, contributing to both global nutrition and the fight against chronic diseases. However, to fully harness this potential, challenges associated with food safety, allergenicity, and public perception will need to be addressed, ensuring that edible insects are accepted as safe, environmentally friendly, and nutritious food sources.

Several literature reviews have explored the nutritional and safety aspects of edible insects; however, few have specifically examined their functional effects on health. Most focus on broader topics such as food security, sustainability, and general nutrition. Currently, no up-to-date review comprehensively summarizes both the functional benefits and safety concerns of edible insects. This review aims to bridge that gap by providing an in-depth analysis of their bioactive compounds, potential health benefits—including effects on the cardiovascular, immune, and digestive systems—safety considerations such as microbial contamination and allergenicity, and regulatory frameworks ensuring their safe consumption. By compiling the most recent and relevant findings, this work offers a comprehensive perspective on the potential of edible insects as functional foods while addressing key safety considerations.

## 2 Edible insects as sources of bioactive compounds

### 2.1 Edible insects as sources of (poly)phenolic compounds

Plant-derived phenolic compounds (also known as phytochemicals) are secondary metabolites characterized by their structure, which includes one or more aromatic rings with a minimum of one hydroxyl group attached. These phytochemical compounds are categorized according to their chemical nature and structure as either flavonoids or non-flavonoids (11). In plants, they perform important biological functions, including defense against microbial and viral pathogens, protection against herbivores, mitigation of the oxidative effects of ultraviolet light, and influencing coloration and fragrance to attract pollinators (12).

**Figure 1:**
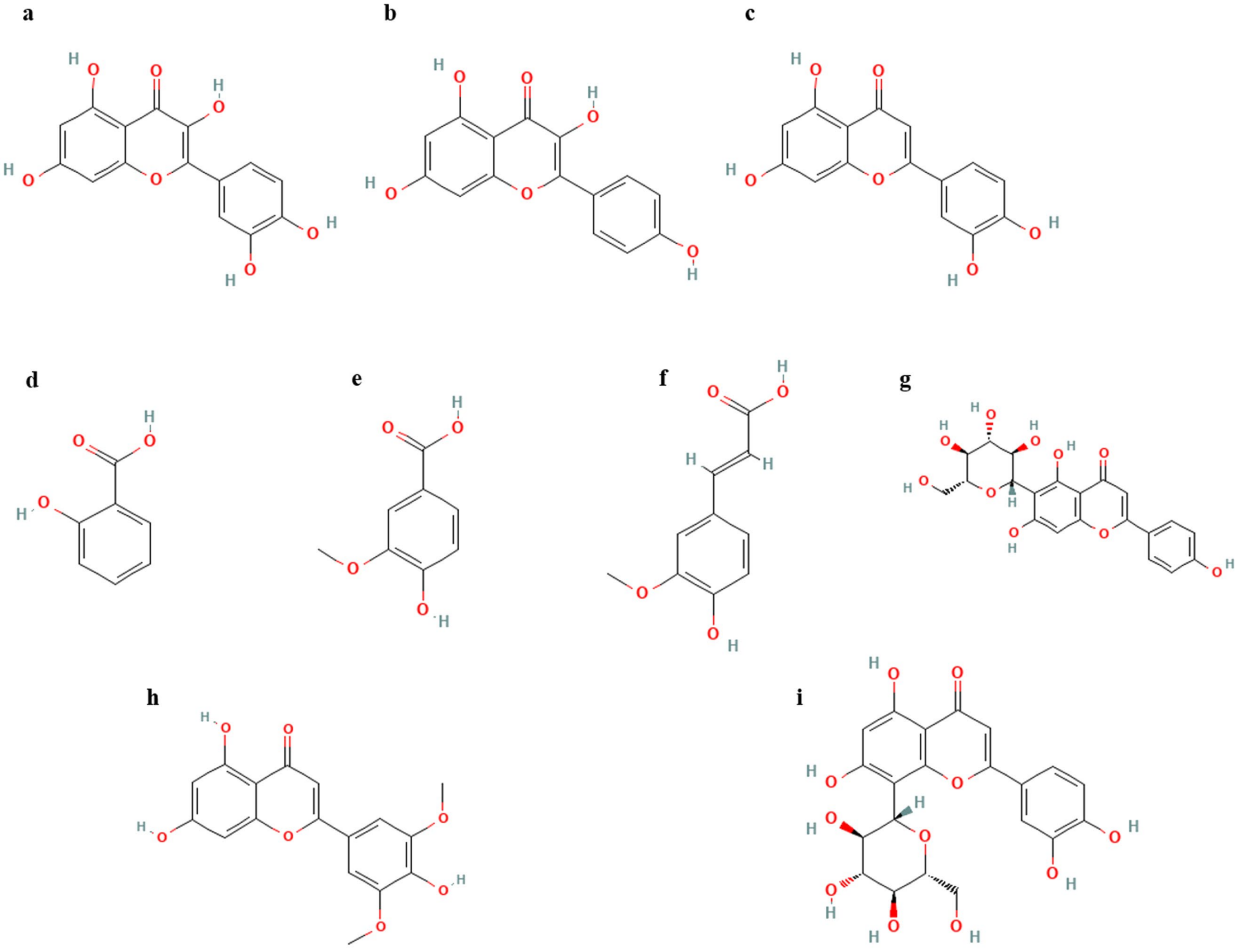
(Poly)phenols reported in various insect species. **(a)** Quercetin, **(b)** Kaempferol, **(c)** Luteolin, **(d)** Salicylic acid, **(e)** Vanillic acid, **(f)** Ferulic acid, **(g)** Isovitexin, **(h)** Tricin, **(i)** Orientin.

Phenolic compounds are widely recognized for their biological effects, including antioxidant properties, modulation of the inflammatory response and its adverse effects, and antimicrobial and anticancer activities (11). These bioactivities make them beneficial to human health when consumed as part of a healthy diet, further enhancing their value as important nutritional components. Given the biochemical versatility of phenolic compounds, previous authors have hypothesized that herbivorous insects consuming plant material assimilate and retain some of these bioactive molecules. In fact, research conducted in the mid-20^th^ century identified phenolic compounds in various parts of insects, such as their exoskeletons and wings. This finding has led to the hypothesis that these phytochemicals, primarily derived from their diet, are absorbed and/or metabolized and subsequently incorporated into their tissues (Table 1; Figure 1). However, the extent to which edible insects can serve as reliable sources of phenolic compounds remains an area that requires further investigation. Understanding their role could significantly enhance knowledge of the nutritional and health benefits associated with entomophagy.

**Table 1:** Phenolic compounds identified across various insect species.

Insect Species	Identified (Poly)phenolic Compounds	Ref.
Adonis blue (*Polyommatus bellargus*)	Isovitexin, Isovitexin-2’-O-xyloside, Kaempferol and quercetin glycosides	(22)
*Amauronematus amplus, Arge* sp.*, Nematus alpestris, Nematus bre-vivalvis, Nematus pravus, Nematus viridis,* and *Trichiosoma scalesii*	Kaempferol and Quercetin glycosides	(116)
American grasshopper (*Schistocerca americana*)	Luteolin (3′,4′,5,7-tetrahydroxyflavone), −β-3-O-glucoside	(117)
*Antheraea pernyi*	Protocatechuic acid, Quercetin 3-O-glucoside, Isoquercitroside, Kaempferol 3-O-glucoside, Kaempferol 3-O-galactoside (trifolin), Tricin 4′-O-(β-guaiacylglyceryl) ether 7-O-hexoside, Tricin 4′-O-(β-guaiacylglyceryl) ether O-hexoside, Tricin 7-O-hexoside Luteolin 7-O-glucoside, Luteolin 6-C-glucoside, Orientin, Luteolin C-hexoside, C-hexosyl-luteolin O-p-coumaroylhexoside and Hesperetin 5-O-glucoside	(118)
Carolina grasshopper or Carolina locust (*Dissoteira carolina*)	Quercetin and Quercetin-β-3-O-glucoside	(25)
Carolina locust (*Dissosteira carolina*)		
Chinese black ant (*Polyrhachis vicina* Roger)	Salicylic acid, trans-cinnamic acid, vanillic acid, isoferulic acid, gallic acid, 3,4-dihydroxybenzoic acid, and caffeic acid, Formononetin, Liquiritigenin, naringenin, sakuranetin, Quercetin, Catechin, L-epicatechin	(119)
Pánfila (*Coenonympha pamphilus*)	Tricin (4′,5,7-trihydroxy-3′,5′-dimethoxyflavone)	(18)
Common blue butterfly (*Polyommatus icarus*)	Quercetin, Kaempferol, Kaempferol 3-O-glucoside, Kaempferol 3-O-(6′-malonyl) glucoside, Kaempferol 3-O-galactoside, Kaempferol 3,7-O-diglucoside, Kaempferol-3-O-rhamnoside, Quercetin 3-O-glucoside, Quercetin 3-O-galactoside, Quercetin-3-O-rhamnoside, Myricetin-3-O-rhamnoside	(19, 20, 27, 120)
Dark black chafer (*Holotrichia parallela*)	Resveratrol, Ferulic acid, gallic acid, and protocatechuic acid, Protocatechualdehyde, 4-Hydroxyacetophenone, Epicatechin, Catechin and Quercetin	(120)
Dark black chafer beetle (*Holotrichia parallela*)	Catechin	(121)
Edible ground cricket (*Henicus whellani*)	Kaempferol and quercetin, Tannins	(122)
Halkhill blue butterfly (*Lysandra coridon* Poda)	Kaempferol, Kaempferol 7-rhamnoside, Kaempferol 3-rhamnoside, Kaempferol 3-glucoside, Quercetin 3-glucoside, Isorhamnetin 3-glucoside, Kaempferol 3-glucoside, 7-rhamnoside, Quercetin 3,7-diglucoside, Isorhamnetin 3,7-diglucoside	
House cricket (*Archeta domesticus*)	Gallic acid, 4-hydroxybenzoic acid, chlorogenic acid, caffeic acid, syringic acid, p-coumaric acid, ferulic acid, and sinapic acid, 2-hydroxybenzoic acid, Daidzein Quercetin, Naringenin and Apigenin	(123)
Large white butterfly (*Pieris brassicae*)	Kaempferol-3-O-sophoroside-7-O-glucoside, Kaempferol-3-O-sophoroside, Ferulic and Sinapic acids	(124, 125)
*Lysandra coridon Poda*	Kaempferol-glycosides	(126)
Marbled white butterfly (*Melanargia galathea*)	Tricin, Tricin 7-glucoside, Tricin 7-diglucoside, Tricin 4′-glucoside, Luteolin, Luteolin 7-glucoside, Luteolin 7-diglucoside, Luteolin 7-triglucoside, Apigenin, and Apigenin 7-glucoside, Orientin, Orientin 7-glucoside, Iso-orientin, Iso-orientin 7-glucoside, Vitexin, Vitexin 7-glucoside, Isovitexin, and Isovitexin 7-glucoside	(127, 128)
*Melanargia galathea*	Tricin (4′,5,7-trihydroxy-3′,5′-dimethoxyflavone), Apigenin (4,5,7-trihydroxyflavone), Tricin 7-glucoside, and Tricin 4′-conjugate, Orientin (8-glucosylluteolin), Orientin 7-glucoside, Isoorientin (luteolin 6-C-glucoside), Isovitexin (6-C-glucosylapigenin), Vitexin 7-glucoside, Luteolin 7-diglucoside	(123)
*Melanargia galathea*	Tricin (4′,5,7-trihydroxy-3′,5′-dimethoxyflavone), Lutexin, Glycosides of tricin (tricin-glycosides)	(128)
Mulberry white caterpillar (*Rondotia menciona*)	Quercetin-glycosides, Kaempferol-glycosides	(23)
Mulberry white caterpillar (*Rondotia menciana*)	Quercetin 3-O-galactosyl-galactoside, Quercetin-3-O-galactoside, Kaempferol 3-O-galactosyl-galactoside, Kaempferol 3-O-galactoside, Quercetin 3-O-β-D-galactopyranosyl-(1 → 3)-β-D-galactopyranoside, and Kaempferol 3-O-β-D-galactopyranosyl-(1 → 3)-β-D-galactopyranoside	(23)
*Neodiprion sertifer*	(+)-Catechin 7-O-β-glucoside, Isorhamnetin 3,7,4′-tri-O-β-glucoside, Kaempferol 3,7, 4′-tri-O-β-glucoside, Kaempferol 3,7, 4′-tri-O-β-glucoside, and Quercetin 3,7,4′-tri-O-β-glucoside	(25)
*Pieris brassicae*	Kaempferol glycosides, Ferulic and sinapic acids	(129)
Silkworm (*Bombyx mori*)	Quercetin, Kaempferol, Quercetin 5-O-β-D-glucoside, Quercetin 7-O-β-D-glucoside, Quercetin 4’-O-β-D-glucoside, Quercetin 5-glucoside, Quercetin 5,4-diglucoside, Quercetin 5,7,4′-triglucoside, Kaempferol 5-O-β-D-glucoside, and Kaempferol 7-O-β-D-glucoside,	(24, 130)
Stink Bugs (*Tessaratoma papillosa*)	Gallic acid, Protocatexhuic acid, p-hydroxybenzoic acid, Vanillic acid, Syringic acid, Vanillin, Ferulic acid, Sinapic acid, and Cinnamic acid, Genistic acid, Rutin, Quecetin, Apigenin, Kaempferol, Myricetin	(136)
White caterpillar (*Rondotia menciana*)	Quercetin 3-O-β-D-galactopyranoside, and Kaempferol 3-O-β-D-galactopyranoside	(23)

### 2.2 Sclerotisation as source of phenolic compounds in insects

Sclerotization is a vital biological process responsible for the hardening and stabilization of the insect cuticle, enabling it to function as both a structural framework and a protective barrier. This process occurs through the integration of phenolic compounds into the cuticular matrix, where they interact with structural proteins and chitin in enzyme-mediated reactions (13). While dietary intake contributes to the availability of phenolics in insects, recent studies have demonstrated that many of these compounds are synthesized endogenously through complex biochemical pathways (14). The cuticle, a multifunctional component of insect anatomy, is composed of two main layers: (i) the procuticle, consisting of chitin in the form of fine filaments that are embedded within a protein matrix, which provides strength and flexibility, and (ii) the epicuticle, composed of proteins and lipids, which acts as a barrier against desiccation and pathogens. The rigidity of the cuticle limits growth, necessitating periodic molting. During each molt, insects synthesize a new cuticle that undergoes sclerotization to acquire the mechanical properties needed for survival and functionality. It has been suggested that the biochemical pathway of sclerotization begins with tyrosine, a precursor that is enzymatically converted into acyldopamine derivatives, for instance N-acetyl dopamine (NADA) and N-β-alanyl dopamine (NBAD). These intermediates are subsequently oxidized to quinones, which serve as reactive intermediates. Quinones undergo enzymatic rearrangement to produce unsaturated catechols, including α,β-dehydro-NADA. These catechols, in turn, are oxidized into unsaturated quinoid derivatives, which react with amino acid residues and other catechols in cuticular proteins, forming extensive cross-links. These cross-links are critical for imparting mechanical stability and resistance to the cuticle (15, 16). Research has provided insights into the diversity of compounds involved in sclerotization. For example, it is now evident that the chemical pathways can vary significantly among insect species, involving additional catechols beyond NADA and NBAD (16). The bioavailability and potential applications of these phenolic compounds in insects have also become a topic of interest. Phenolics play a role not only in the structural integrity of the insect cuticle but also in defense mechanisms, such as antimicrobial activity. Improvements in analytical methodologies, which include high-performance liquid chromatography (HPLC) and mass spectrometry, have facilitated a more precise characterization of these compounds, offering valuable insights into the phytochemical profile of insects and their potential biological activities. Despite these advances, gaps remain in the understanding of sclerotization. The specific functions of less-studied catechols, the regulation of enzymatic pathways throughout developmental stages, and the interaction of phenolic compounds with other biomolecules require further investigation. Furthermore, research on how processing methods (e.g., drying or heating) affect the stability and functionality of these phenolics in edible insects is fundamental for their utilization in the food and nutraceutical industries.

### 2.3 Relationship between diet and (poly)phenolic composition in insects

Research has shown that herbivorous feeding behavior is essential for determining insects’ phenolic profile. Insects interact with a diverse range of plant phenolics, with flavonoids being among the most frequently absorbed compounds (17). Since the early 20th century, the metabolism of dietary phenolics in insects has been of significant interest. In 1964, was described the pigmentation of the wings of *Melanargia galathea*, commonly known as the mottled white butterfly, attributing it to flavonoids and suggesting that these compounds function as UV protectors. The authors identified the O-methylated flavone tricin in butterfly wings, providing evidence that insects absorb these compounds from their diet and potentially metabolize them into unique derivatives (18). More recent studies have confirmed and expanded our understanding of insect phenolic composition. It was demonstrated that *Polyommatus icarus*, the common blue butterfly, takes up flavonoids from the plants it feeds on as a larva and utilizes these pigments to contribute to its wing coloration upon reaching adulthood (19). These results are consistent with previous assertions concerning this insect, where it was demonstrated that *P. icarus* exhibits a phenolic profile mirroring that of its host plants, such as *Coronilla varia* and *Medicago sativa*, during its development. Chemical analyses revealed elevated levels of flavonoids in their glycosylated form, such as kaempferol-3-O-glucoside, suggesting that this compound could result from the biotransformation of flavonols (e.g., kaempferol) or their glycoside derivatives from plants within the insect, particularly in early developmental stages like the larval phase (20). Interestingly, these transformations appear to be selective, with kaempferol and its glucosides being preferred over other flavonoids such as quercetin and myricetin. In fact, it has been suggested that glucoside formation represents a typical metabolic process involved in flavonoid metabolism within these organisms (21). This selective uptake and transformation underscore the metabolic adaptability of insects and their ability to enhance the bioavailability of dietary phenolics. Subsequent studies by these authors examined the flavonoid composition in blue butterfly individuals raised on various host plants, with one group feeding on their natural food sources while the other was exposed to non-native hosts or plant parts (19). The findings revealed a direct correlation between the insects’ flavonoid content and their diet, emphasizing the significant impact of host plants. Larvae demonstrated the ability to absorb phenolics from non-natural host plants, albeit in lower quantities compared to those reared on their natural hosts. Additionally, selective uptake of quercetin and kaempferol was noted, with female individuals consistently showing a higher capacity for flavonoid absorption. An investigation into the different life stages of the common blue butterfly (larval, pupal, and adult) reared on vetch (*Pisum sativum*) and alfalfa (*Medicago sativa*) revealed a flavonoid profile closely resembling that of their respective host plants, with kaempferol glycosides (−3-O-glucoside) identified as the predominant phytochemical. Furthermore, an additional kaempferol glycoside (−3,7-di-O-glucoside) was identified during the larval stage, suggesting that it may be a biometabolite derived from flavonols or their glycosylated derivatives present in plants. The finding indicated a preference for the uptake of flavonols and their glycosides (e.g., kaempferol and its derivatives) over other flavonoids (e.g., quercetin and myricetin) also found in these primary plant sources. When larvae consumed a synthetic diet supplemented exclusively with flavonols (e.g., kaempferol), further analyses confirmed the occurrence of kaempferol glycosides (−3-O-glucoside), highlighting the insect’s capacity to conjugate these compounds with sugar molecules (19). Studies on the Adonis blue (*Polyommatus bellargus*) and other Lepidoptera have further revealed the ability of insects to modify dietary phenolics through bioconversion pathways, producing flavonoid derivatives not found in plants. For instance, isovitexin absorbed from *Coronilla varia* is transformed into isovitexin-2’-O-xyloside, indicating sophisticated metabolic processes (22). Beyond Lepidoptera, flavonoid metabolism has been documented in other insects such as the Carolina locust (*Dissosteira carolina*) and the silkworm (*Bombyx mori*). These studies highlight the broad ability of insects to accumulate, metabolize, and integrate phenolic compounds originating from plants into various body structures, including tissues, wings, and cocoons (23, 24). In this context, the analysis of the yellow-colored compounds presents in the wings of the *Carolina locust* revealed the presence of flavonols, specifically a free flavonol of quercetin and its glycoside derivative (−3-β-O-glucoside). These compounds were confirmed to stem from the intake of quercetin derivatives occurring in plant components (25). Studies in this field have detected flavonoids and other phytochemicals in silkworm cocoons, suggesting that the species’ absorption of these plant-derived compounds and their biochemical transformation persist throughout development. Comparable research has detected flavonoid compounds within the silk casing of *B. mori*, illustrating the assimilation of these bioactive molecules from host plants and their later biochemical transformation (21, 26). A comparable pattern was noted in white caterpillar (*Rondotia menciana*) cocoons, which were exclusively reared on mulberry (*Morus alba*) leaves. Two flavonol glycosides, namely quercetin 3-O-β-D-galactopyranosyl-(1 → 3)-β-D-galactopyranoside and kaempferol 3-O-β-D-galactopyranosyl-(1 → 3)-β-D-galactopyranoside, were identified, together with four additional flavonoids. Interestingly, these glycosylated flavonols were absent in the foliage of *Morus alba*, indicating that the insect transformed dietary flavonoids into compounds incorporated into the cocoon (23).

Beyond variations in flavonoid accumulation during different stages of development, differences in flavonoid accumulation between sexes have also been studied, suggesting possible biological functions. Female specimens of butterflies generally exhibit elevated flavonoid levels compared to males, a factor that may influence mate selection behavior (27). Such sexual dimorphism highlights the potential signaling and structural functions of flavonoids in insect wings. This research found that adult female *P. icarus* individuals contained higher levels of flavonoids compared to males when fed *Vicia villosa* (fodder vetch or hairy vetch). The phenolic profile of fully developed fourth-instar caterpillars and adult individuals revealed three glycosides (−3-O-rhamnoside) of the primary flavonols quercetin, myricetin, and kaempferol, all derived from fodder vetch. In adult individuals, a selective uptake of kaempferol glycosides (kaempferol-3-O-rhamnoside) was observed, with only trace amounts of myricetin glycosides (myricetin-3-O-rhamnoside) detected. Female individuals demonstrated a greater ability to accumulate flavonoids, which were predominantly concentrated in their wings. This increased accumulation of flavonoids (e.g., myricetin, quercetin, and kaempferol derivatives) seems to affect the behavior of males during mate selection, indicating a tendency to choose females with higher levels of these compounds (19).

### 2.4 Main polyphenols identified in insects

The above findings highlight that insects contain phenolic compounds. This is particularly true in Lepidoptera species, stemming from their capacity to absorb dietary phenolics and modify these compounds metabolically for integration into their tissues. Insects show a preference for selectively absorbing flavonols such as kaempferol and quercetin, along with flavones like tricin and isovitexin. These phytochemicals are typically glycosylated with a single sugar molecule, such as glucose, rhamnose, or galactose. Widely reported flavonoids include quercetin, kaempferol, luteolin, tricin, apigenin, and myricetin, along with their glycosylated derivatives. Similarly, phenolic acids have been reported in several insect families. Complex glycosides, including isovitexin-2′-O-xyloside and tricin 7-glucoside, as well as catechins such as (+)-catechin and L-epicatechin, further highlight the biochemical diversity in insect tissues and cocoons (28). These phenolic and flavonoid compounds, essential for insect survival, provide structural support to the cuticle, protect against pathogens, and enhance their adaptability to environmental stresses.

Within insect families, the primary phenolic compounds identified are as follows:

- Lepidoptera (e.g., butterflies and moths): Dominant phenolic compounds include flavonoids such as quercetin, kaempferol, luteolin, and their glycosylated forms, as observed in species like *Polyommatus bellargus* (Adonis blue) and *Pieris brassicae* (large white butterfly). These compounds often appear as glycosylated derivatives, enhancing their functional roles.
- Orthoptera (e.g., grasshoppers and crickets): Species such as *Schistocerca americana* (American grasshopper) and *Henicus whellani* (edible ground cricket) primarily feature luteolin, kaempferol, and quercetin derivatives, reflecting their adaptation to varied dietary sources.
- Hymenoptera (e.g., ants): Phenolic profiles of *Polyrhachis vicina* (Chinese black ant) include hydroxybenzoic acids like salicylic acid and vanillic acid, along with flavonoids, including quercetin and catechins, showcasing their metabolic complexity.
- Coleoptera (e.g., beetles): Species like *Holotrichia parallela* (dark black chafer beetle) are rich in catechins, resveratrol, and protocatechuic acid, highlighting their ecological interactions with plant-derived secondary metabolites.

This classification underscores the diversity of phenolic compounds across insect families and their ecological and functional significance. Table 1 summarizes the distinct groups of (poly)phenolic compounds identified in various insect species, as reported by different authors.

## 3 Potential health benefits of edible insects

Beyond their nutritional potential, edible insects are likewise acknowledged for their positive effects on health, including immunomodulatory, antioxidant, antimicrobial, and beneficial effects on cardiovascular function, as well as positive effects on the gastrointestinal system, primarily associated with their diverse array of bioactive and functional compounds.

### 3.1 Immunomodulatory effects of compounds derived from edible insects

Several studies have demonstrated the anti-inflammatory and immunomodulatory effects of compounds derived from edible insects. Ahn and colleagues extensively studied glycosaminoglycan, a polysaccharide present in crickets, and its beneficial effects using rodent models (29, 30). Their research revealed that glycosaminoglycan extracted from *Gryllus bimaculatus* displayed significant anti-inflammatory effects in rats suffering from chronic arthritis. This was achieved through the inhibition of C-reactive protein (CRP) and rheumatoid factor, along with the suppression of several inflammatory biomarkers *in vitro*. Moreover, the simultaneous administration of glycosaminoglycan and indomethacin, a nonsteroidal anti-inflammatory agent, proved to be more effective in alleviating paw edema than either treatment used independently (29). Another study looked at daily intake of a processed (dried and roasted) cricket powder (25 grams) in adult volunteers and found an increase in the beneficial bacteria *Bifidobacterium animalis* along with a reduction in plasma concentrations of tumor necrosis factor alpha (TNF-α) (31). Moreover, the ingestion of chitooligosaccharides, which are depolymerized forms of chitin and chitosan, over an 8-week period markedly decreased the levels of pro-inflammatory cytokines TNF-α and interleukin-1β (IL-1) in older adults (32). Furthermore, it was also demonstrated that dietary supplementation with chitin or chitosan in fish enhanced immune function by increasing white blood cell count, hemoglobin levels, and red blood cell count (33). A water-based extract derived from green beetles (*Mimela* sp.) was shown to boost the humoral and cellular immune functions, reduce histopathological damage, increase leukocyte counts, and enhance cytokine production of TNF-α and IL-6 of immunosuppressed mice (34). Moreover, studies utilizing supercritical CO₂ extracts from yellow mealworm larval powder showed enhancements in two types of immune responses (cellular and humoral) in female Kunming mice (35). Thirteen nitrogen-based non-peptide compounds (primarily identified as alkaloids) isolated from Weaver ants (*Polyrhachis dives*) exhibited *in vitro* anti-inflammatory, immunosuppressive, and renoprotective effects (36). Furthermore, the immunomodulatory role and biochemical pathways of two purified polypeptide components isolated from bee pupae were investigated *in vivo* and *in vitro*. These compounds markedly enhanced body weight gain rates, organ coefficients, macrophage phagocytic activity, delayed hypersensitivity reactions, cytokine levels (interleukin [IL]-2 and interferon [IFN]-γ), and immunoglobulin concentrations (IgA, IgG, and IgM) and standard hematological parameters in immunosuppressed mice treated with cyclophosphamide. Functional analyses using RAW264.7 cells demonstrated that one of these compounds promoted cytokine release (IL-2, tumor necrosis factor-α, and IFN-γ) and nitric oxide synthesis by augmenting the expression of corresponding mRNA. This compound also displayed immunoregulatory effects by enhancing ERK and p38 phosphorylation and influencing the expression of nuclear transcription factors (EIK-1, MEF-2, and CREB) within the MAPK signaling cascade (37).

### 3.2 Antioxidant capacity of compounds derived from edible insects

Antioxidants play a crucial role in maintaining human health by neutralizing harmful free radicals that contribute to oxidative stress, a key factor in aging and the development of chronic diseases such as cardiovascular disorders, neurodegenerative conditions, and cancer. These bioactive compounds help protect cells from damage, support immune function, and promote overall well-being (38). In recent years, edible insects have gained attention as a sustainable source of antioxidants, with studies highlighting the presence of bioactive peptides, polyphenols, and other antioxidant compounds that may contribute to their potential health benefits. Numerous studies have emphasized the antioxidant properties associated with different extraction techniques and components derived from edible insects. For instance, recent research demonstrated that aqueous extracts of the green beetle (*Mimela* sp.) exhibited significant *in vivo* antioxidant effects in mice with cyclophosphamide-induced immunosuppression (34). Similarly, both water- and lipid-soluble extracts from various insects, black ants (*Lasius niger*), house crickets (*A. domesticus*), grasshoppers, mealworms, buffalo worms (*Alphitobius diaperinus*), silkworms, African caterpillars (*Imbrasia oyemensis*), palm worm larvae (*R. ferrugineus*), giant water bugs (*Lethocerus indicus*), evening cicadas (*Tanna japonensis*), and *Scolopendra gigantea* (centipede), have demonstrated notable antioxidant effects (39). The researchers discovered that aqueous extracts from crickets, grasshoppers, and silkworms displayed the highest antioxidant potential (TEAC), exceeding that of fresh orange juice by fivefold. Conversely, extracts from evening cicadas, giant water bugs, black scorpions, and Thai zebra tarantulas showed only marginal antioxidant activity. Regarding the lipid-soluble fractions, silkworms, evening cicadas, and African caterpillars displayed the highest TEAC levels, approximately double that of olive oil. Conversely, Thai zebra tarantulas, palm worms, and black ants ranked lowest in antioxidant activity (39). Similarly, extracts from yellow mealworms (*T. molitor*) and beetles (*Ulomoides dermestoides*), which have abundant saponins, carbohydrates, and proteins, demonstrated enhanced antioxidant activity *in vitro* (40). Ethanol and ethanol-aqueous extracts from *A. domesticus* and *T. molitor* demonstrated notable antioxidant potential, which showed a strong correlation with their overall phenolic composition (41, 42). Meanwhile, research employing the *Caenorhabditis elegans* within an *in vivo* model revealed that peptides obtained from the tropical banded cricket (*Gryllodes sigillatus*) efficiently lowered reactive oxygen species (ROS) levels and extended the lifespan of the worms reared in both acute and chronic oxidative stress scenarios (43). Likewise, the use of silkworm larvae (*B. mori*) powder in a Drosophila model was found to extend the lifespan of the flies, showing greater longevity compared to those maintained on a standard diet (44). Certain food processing methods have been also found to positively influence the chemical composition and antioxidant activity of insect-derived products. For example, the fermenting of insects and insect-derived flours has been found to generate bioactive metabolites that boost their antioxidant potential. A prominent case is using *Lactococcus lactis* strains to ferment flours made with yellow mealworms and grasshoppers (*Sphenarium purpurascens*), which markedly enhanced their antioxidant capacity (45).

### 3.3 Antimicrobial effects of compounds derived from edible insects

The potential antibacterial effects of specific bioactive compounds obtained from edible insects have also been studied. In particular, extracts from yellow mealworms and beetles (*Ulomoides dermestoides*), abundant in saponins, carbohydrates, and proteins, have demonstrated antimicrobial effects against both Gram-negative bacteria, such as *Shigella flexnerii* and *Proteus vulgaris*, as well as Gram-positive bacteria, including Bacillus spp. (40). Moreover, chitin and chitosan extracted from house crickets and tropical banded crickets have exhibited strong antibacterial effects against *Escherichia coli* ATCC 25922 and *Listeria innocua* ATCC 33090 (46). A study documented the antibacterial effects of lauric acid, a medium-chain saturated fatty acid (MCFA), extracted from black soldier flies in young pigs (47). Their findings revealed that lauric acid helped protect the piglets from bacterial infections and effectively controlled Gram-positive bacteria in their gastrointestinal tracts. Additionally, the antibacterial effectiveness was notably increased when lipase was added to the piglets’ diet along with the black soldier fly-derived supplement (47).

### 3.4 Cardioprotective effects of bioactive compounds from edible insects

Compounds isolated from edible insects have also demonstrated potential in preventing cardiovascular diseases. The existence of unsaturated fatty acids in insects such as mealworms, crickets, and housefly larvae might contribute to lowering the risk of cardiovascular disease (48). In fact, a recent study found that mealworm, mulberry silkworm, and long-horned grasshopper are rich sources of unsaturated fatty acids, making up 73.4, 68.6, and 63.7% of their total lipid content, respectively. Among these, the mulberry silkworm had the highest omega-3 content, accounting for 13.7% of its total lipids (49). Meanwhile, the quality analysis of oils and flours derived from the crickets *Scapsipedus icipe* Hugel, Tanga, and *Gryllus bimaculatus* De Geer from Africa revealed that palmitic acid was the predominant saturated fatty acid, while oleic acid was the dominant monounsaturated fatty acid (50). The beneficial fatty acid profile of the chontacuro—or edible Amazonian weevil larva (*Rhynchophorus palmarum* L.)—from the Ecuadorian Amazon was also recently reported. Oleic acid was identified as one of its main fatty acids, as was palmitic acid (51). Bioactive peptides derived from tropical *G. sigillatus* have been reported to exhibit antidiabetic and antiglycemic effects in *in vitro* models (52, 53). Similarly, chitin and chitosan isolated from *A. domesticus* and *G. sigillatus* have shown hypolipidemic effects (46). Moreover, ethanol and ethanol-water polyphenol extract from house crickets and mealworms have demonstrated antiobesity effects (41). Another study on malnourished male mice found that incorporating tropical cricket (*G. sigillatus*) powder into their diet resulted in increased body weight and a reduction in serum triglycerides (54). Additional research has shown that consuming *T. molitor* larvae powder every day produces anti-obesity effects *in vivo* when tested on high-fat diet-induced obese mice. This was evident by observing a reduction in body weight gain and a decrease in visceral fat mass (55). Similarly, silkworm oil demonstrated anti-dyslipidemic effects in a Drosophila *in vivo* model (44).

Fermentation also affects the cardioprotective potential of insect-derived components. One study demonstrated that fermenting flours made from yellow mealworms and grasshoppers (*Sphenarium purpurascens*) using *Lactococcus lactis* strains significantly enhanced their antihypertensive effects (45). In rats subjected to a high-fat diet, glycosaminoglycan extracted from *G. bimaculatus* crickets was found to lower CRP levels, abdominal and epididymal fat accumulation, along with several sero-biochemical parameters, such as phospholipids, aspartate aminotransferase, alanine aminotransferase, total cholesterol, and glucose. These findings suggest that glycosaminoglycan could have potential applications in preventing conditions such as fatty liver disease or hyperlipidemia (30). Additionally, research on diabetic mice receiving glycosaminoglycan supplementation revealed improvements in metabolic parameters. Mice subjected to treatment showed decreased blood glucose and LDL-cholesterol levels, along with increased activity of certain antioxidant enzymes such as catalase, superoxide dismutase, and glutathione peroxidase (56). These outcomes suggest that cricket-derived glycosaminoglycan may contribute to reducing the likelihood of developing diabetes and chronic inflammatory conditions.

### 3.5 Gut health benefits of insect-derived compounds

The beneficial effect of different insect-derived compounds on gastrointestinal health has also been demonstrated. The beneficial effects of supplementing healthy adult individuals with 25 grams per day of dried, roasted cricket powder over a 14-day period were recently reported. The intervention led to an increase in the relative abundance of *Bifidobacterium animalis*, a probiotic bacterium. This particular probiotic, known for its role in preventing respiratory infections and diarrhea as well as mitigating the side effects of antibiotics, was more prevalent after the supplementation, indicating that cricket-based diets may support gastrointestinal health through beneficial changes that occur in the gut microbiota (31). Researchers have also observed enhanced microbial diversity in rainbow trout that consumed black soldier fly larvae. The increased microbial diversity in their gut enhanced resilience by supporting competition with pathogens for resources and attachment sites (57). Moreover, probiotics obtained from 0.4% dried mealworm and super mealworm larvae were able to effectively mitigate *E. coli* and Salmonella infections in broiler chickens (58). Insects’ beneficial effects on gut microbiota and their antimicrobial properties have been linked to their chitin content. This polysaccharide is made up of *β*-1,4-N-acetylglucosamine and serves as a fundamental structural element of insects’ exoskeletons (31, 59). For instance, cricket powder contains dietary fiber levels comparable to those of whole wheat and soy powder, with all its fiber being derived from chitin (56).

Besides supporting the proliferation and variety of beneficial gut microbes, insect-based diets are also linked to the production of short-chain fatty acids (SCFAs). In chickens, the chitin was broken down by gut microbiota, resulting in SCFA production, e.g., propionate and butyrate (59). These SCFAs promote the secretion of hormones that control satiety, meaning that chickens on an insect-based diet consume less feed than those on a soybean-based diet. Higher concentrations of propionate and butyrate are also connected to lower blood cholesterol and triglyceride levels, as well as enhanced energy metabolism in chickens fed insect-derived meals (59).

## 4 Safety considerations for edible insects

The safety and harmlessness of edible insects as a newly introduced nutritious food and their applications in food products have become a significant concern for consumers. Food industry researchers are actively investigating possible biohazards and chemical risks to determine whether edible insects pose significant threats to human health. The microbiological quality and antinutritional properties of edible insects are among the most critical safety considerations. Other factors, such as potential contamination with chemical residues, toxic metals, fungal toxins, and allergenic compounds, must also be carefully evaluated to ensure their safety and suitability for consumption (3).

### 4.1 Heavy metals

Plants can incorporate heavy metals into their tissues from the soil and through exposure to air pollution. This accumulation of heavy metals in plant tissues can subsequently be transferred to herbivorous insects that feed on them. As a result, plant products such as nectar, leaves, flowers, and fruits, as well as the organisms that consume them, including insects, can become contaminated and accumulate these metals (60). Among the heavy metals most frequently transferred from plants to insects, cadmium and lead are particularly prominent (60). The presence of cadmium (Cd) in edible insects, including yellow mealworms, giant mealworms, grasshoppers, locusts, termites, and black soldier fly larvae, has been widely documented (1, 61). The ability of the black soldier fly (*Hermetia illucens*) to accumulate Cd is well established (62). Previous analyses of Cd content in black soldier flies reported concentrations of up to 1.68 mg/kg in larvae, 0.94 mg/kg in pupae, and 0.50 mg/kg in adult flies (63). Studies on the impact of heavy metal pollution on Cd accumulation in black soldier fly larvae indicated that Cd from the substrate was retained in the larvae (64). These findings align with studies on black soldier fly prepupae reared on food and fruit waste, as well as poultry manure, where traces of Cd were detected at low concentrations (0.03–0.16 mg/kg) (63). Similarly, in yellow mealworms larvae, Cd traces were found at concentrations up to 0.016 mg/kg, likely derived from their feeding substrate (65). Other edible insect species have also been found to contain Cd. For example, in four selected edible insect species, Cd concentrations did not exceed 0.72 mg/kg (66). Despite these findings, in all cases, Cd concentrations remained within permissible limits, making these insects a viable alternative for animal feed without posing contamination risks or compromising the safety of livestock-derived food products. However, other studies have reported less favorable results, with significantly higher Cd concentrations detected in black soldier fly larvae and prepupae, reaching up to 2.79 mg/kg in larvae and 2.94 mg/kg in prepupae (67, 68). Additionally, Cd levels as high as 9.1 mg/kg have been recorded in black soldier fly larvae (64). These findings are consistent with previous studies on the same insect species, which reported Cd concentrations of up to 8.33 mg/kg (69). Research on Cd content in other edible insects, such as grasshoppers, ladybirds, and locusts, has also found elevated Cd concentrations, reaching 7.11 mg/kg, 5.78 mg/kg, and 3.51 mg/kg, respectively (70). These values exceed the recommended threshold of 2–3 mg/kg for animal-derived food products set by China, the FAO/WHO, and the EU Directive (71).

Several studies have also assessed lead (Pb) concentrations in edible insects, indicating that, in most cases, the detected levels fall within permissible limits for both animal and human consumption. Pb concentrations not exceeding 0.079 mg/kg were reported in *T. molitor* larvae developed in diverse substrates (65). In black soldier fly larvae, Pb levels did not exceed 2.68 mg/kg, supporting its viability as a potential substitute for fishmeal in animal feed (72). Similarly, Pb concentrations in prepupae of this species reached up to 0.99 mg/kg, complying with EU standards, while in adult flies, levels were even lower, ranging from undetectable to 0.17 mg/kg (67). In contrast, in termites from Nigeria (*M. bellicosus*, *R. flavipes*, *Kalotermes flavicollis*), no detectable Pb was reported (73). Additionally, Pb levels in termites were recorded at up to 0.03 mg/L. (74) Furthermore, yellow mealworms and superworms (*Zophobas morio*) raised on a diet of wheat and oat derivatives exhibited Pb levels below the detection limit (61). Pb levels reported in other insect species intended for consumption were 0.7 mg/kg in grasshoppers, 1.14 mg/kg in ladybirds, 0.23 mg/kg in termites, and 0.04 mg/kg in locusts (70). Overall, these findings suggest that Pb exposure through insect consumption is minimal and aligns with regulations established by the EU, FAO/WHO, and China.

Research on weevil species (*Rhynchophorus phoenicis*) and large scarab beetles (*Anapleptes trifaciata*) has detected concentrations of nickel, copper, and other potentially harmful metals (cadmium and lead) (75). Similarly, studies on certain edible insects, including *Eligma narcissus* (moths), *Locusta migratoria* (grasshoppers), and *Acrida chinensis* (long-headed grasshoppers), which are part of traditional diets in China, have detected traces of mercury and other heavy metals, such as cadmium and lead (76). Notably, plants collected from the same locations as these insects exhibited lower levels of heavy metals compared to the insects themselves, suggesting bioaccumulation. In South Africa, a study on mopane worms (*Imbrasia belina*) detected elevated levels of cadmium, copper, and manganese, surpassing the maximum allowable limits for human consumption established by the European Commission as well as the U.S. Food and Drug Administration (FDA) by a factor of at least three (77). In contrast, research conducted in Korea on consumable Orthoptera species, specifically *Oxya chinensis formosana*, identified the presence of cadmium, arsenic, lead, and mercury. Although these contaminants were detected, their concentrations remained within the permissible limits set by European regulations (Commission Regulation 1881/2006) for food products (78). Similarly, various insect-derived products have been found to contain heavy metals, including toxic elements like cadmium, arsenic, chromium, and lead. However, these concentrations typically comply with the established safety thresholds for human consumption (79). Recent studies on *R. palmarum* L larvae. Have shown that it contains a wide range of metals, metalloids, and non-metals. However, no evidence of toxic metal elements was observed, suggesting that this species may be a safer alternative for consumption (51). Despite these findings, the possible toxic impact of metallic contaminants in insects intended for consumption should be carefully considered. While many edible insects currently comply with legal safety standards in terms of heavy metal content, ongoing monitoring and risk assessments remain essential to ensure their long-term safety for consumers.

### 4.2 Pesticides

Edible insects, due to their natural behavior, are exposed to various contaminants commonly used in agricultural pest control, including chemical pesticides such as insecticides, herbicides, and fungicides. Contamination can occur directly—through pesticide application or irrigation—or indirectly when insects consume plants or water containing pesticide residues (80).

Several studies have sought to identify pesticide residues in both edible and inedible insects. A study revealed that both hive products and honey bees (*Apis mellifera*) contained residues of insecticides, including chlorpyrifos and chlorfenvinphos, with some samples also showing traces of pesticides banned in the European Union, such as dichlofenthion and fenitrothion (81). An analysis of pesticide residues or presence in black soldier flies, house crickets, and yellow mealworms did not reveal any detectable traces. However, isoproturon was found in grasshoppers (*L. migratoria*) (82). Another study examining flying insects collected throughout Germany identified contamination from 17 different pesticide residues. Although specific insect species were not identified, herbicides such as metolachlor-S, prosulfocarb, and terbuthylazine were frequently detected, along with the insecticide thiacloprid (83). Additionally, studies on wax moths (*Galleria mellonella*), grasshoppers, mealworm beetles, and buffalo worms (*Alphitobius diaperinus*), as well as insect-based products such as insect balls, cricket croquettes, and insect burgers, revealed the presence of pesticides, including methoprene, empenthrin, and pirimiphos-methyl. Herbicides such as chlorbufam and difenzoquat, as well as fungicides like azoxystrobin and cycloheximide, were also detected (84). Despite these findings, contamination levels in edible insects were reported to be below those typically identified in conventional animal-derived products. While the occurrence of pesticide residues in insects intended for consumption does not appear to pose immediate health risks to consumers according to the studies reviewed (82), it remains a concern due to its potential implications for human and animal health.

### 4.3 Mycotoxins

Toxic fungal metabolites are acknowledged as a biological risk in insects intended for consumption, alongside pathogenic microorganisms, heavy metals, and pesticides. These toxic compounds are generated mainly by fungi of the Fusarium, Aspergillus, and Penicillium groups. A number of studies have found the presence of these hazardous compounds in insects. For example, research on edible stink bugs (*Encosternum delegorguei*) detected the human carcinogen aflatoxin B1 in concentrations lower than the threshold recommended by the World Health Organization (WHO) of 20 ng/g for this mycotoxin (85). More encouragingly, the analysis of insect-based products such as house cricket flour, silkworm pupa powder, and black crickets (*G. bimaculatus*) found no detectable levels of mycotoxins at quantifiable concentrations (86). In contrast, significant mycotoxin contamination was reported in yellow mealworms, including alternariol, HT-2 toxin, and roquefortine (82). Additional studies found that grasshoppers contained residues of nicarbazin and nivalenol, house crickets contained alternariol methyl ether, and black soldier flies tested positive for zearalenone. Other studies analyzed aflatoxin levels in dried caterpillars and termites sold in Zambian markets. Aflatoxin was detected in all samples, with concentrations exceeding established limits for both insects. Under simulated high-humidity storage conditions (30°C for 7 days), AF levels increased by at least 20-fold, reaching potentially hazardous levels for human consumption (87). Most studies relied on detection or qualitative methods to identify mycotoxin contamination. This underscores the need for further research focused on quantifying mycotoxin levels in edible insects and assessing their potential health effects on consumers. A substantial gap in understanding persists concerning the prolonged effects of consuming insects intended for consumption that carry chemical and biological risks, reinforcing the need for ongoing research in this field.

### 4.4 Microbiological safety

Edible insects also harbour a diverse range of microorganisms, some of which offer protective benefits to their hosts by defending them from harmful microbes, parasitic organisms, and other threats (88), while others may pose microbiological risks to humans. Like conventional plant- or animal-derived food sources, edible insects could be carriers of pathogenic microorganisms, including bacteria, viruses, protozoa, fungi, rickettsiae, and nematodes (89). The FAO has indicated that most pathogens specific to insects are classified separately from those affecting vertebrates, making them unlikely to pose a risk to humans (90). However, the EFSA highlights that ensuring the safety of insects intended for consumption is largely influenced by the methods used in their cultivation and processing (8). Despite these assurances, consumer concerns regarding the microbial safety of insect-based products persist. A number of studies have assessed the microbial profiles of live edible insects. For example, analyses of microbial levels in *T. molitor* and *Locusta migratoria migratorioides* larvae indicated an elevated microbial presence, including overall aerobic bacteria, members of the Enterobacteriaceae family, lactic acid-producing bacteria, fungi, and yeast (91), all of which exceeded acceptable thresholds for certain ready-to-eat foods (92). Similarly, a study on fresh yellow mealworms, house crickets, and African crickets (*Brachytrupes* sp.) reported total viable counts ranging from 6.7 to 7.7 log CFU/g, along with high levels of Enterobacteriaceae (93). Additionally, a study on fresh grasshoppers found elevated microbial counts, including total aerobic mesophilic bacteria, lactic acid bacteria, *Escherichia coli*, Salmonella, yeasts, fecal coliforms, and total coliforms. A recent study on fresh samples of chontacuro larvae (*R. palmarum* L.) found no evidence of pathogenic microorganisms. The study reported microbial counts for aerobic bacterial spores, yeasts, molds, aerobic mesophilic bacteria, lactic acid bacteria, and Enterobacteriaceae. However, the reported values were within the range of those observed in other edible insects (94).

Despite the elevated microbial content reported in edible insects, cooking and other food processing methods can significantly reduce microbial loads. Common methods involve heat-based processing such as steaming, roasting, frying, smoking, stewing, and braising. These techniques not only improve flavor but also help reduce microbial presence (94). What is more, insects are frequently transformed into fine powders, compressed extrudates, or fully dehydrated products utilizing methods that generally incorporate heat treatments or drying processes—both of which effectively minimize microbial contamination (95). Indeed, minimal microbial levels have been observed in heat-treated insects, including mealworms, house crickets, and African crickets, when subjected to boiling, roasting, or stir-frying, with total viable counts below 5 log CFU/g and Enterobacteriaceae levels under 3 log CFU/g (93). Boiling, in particular, has been highly effective in eliminating Enterobacteriaceae (<1 log CFU/g), whereas roasting has proven less efficient. Other research supports these findings. Frying grasshoppers has been shown to significantly decrease microbial loads, including *Escherichia coli* (approximately 3.6 log CFU/g reduction), Salmonella (around 4.5 log CFU/g), fecal coliform bacteria (about 5.9 log CFU/g), and total coliforms (roughly 4.3 log CFU/g). Processed insect products generally exhibit lower microbial loads compared to fresh insects. The analysis of commercial insect-based food products, including powdered and dried house crickets, locusts (*L. migratoria*), and mealworms, revealed low microbial levels, with mesophilic aerobic bacteria remaining below 5 log CFU/g, lactic acid bacteria below 5 log CFU/g, Enterobacteriaceae under 2 log CFU/g, and yeasts and molds measuring less than 5.1 log CFU/g and 3.1 log CFU/g, respectively (96).

Most studies emphasize the importance of proper cultivation and processing methods to minimize microbial risks. Existing guidelines in both Europe and the USA mandate that insects for human consumption must be farmed in authorized facilities and must not be sourced from the wild. This controlled environment allows for better hygiene management and minimizes contamination risks.

## 5 Allergenicity

Various studies have identified proteins in edible insects that can trigger allergic reactions, some of which share similarities with allergens found in crustaceans and dust mites. This cross-reactivity suggests a potential risk for individuals with pre-existing food allergies. Allergic reactions to edible insects have been reported in species such as silkworms, mealworms, caterpillars, *Bruchus lentis*, sago worms, locusts, grasshoppers, cicadas, bees, *Clanis bilineata*, and carmine, a food additive derived from *Dactylopius coccus* (97). The main allergens identified in insects include tropomyosin and arginine kinase, both classified as pan-allergens due to their structural similarity to proteins in crustaceans and house dust mites, which may lead to cross-reactivity (97). Indeed, previous studies have showed that cricket tropomyosin shares over 60% sequence identity with allergens found in various species of shellfish, insects, and nematodes (98). However, other protein allergens, such as arginine kinase and glyceraldehyde 3-phosphate dehydrogenase, have also been recognized as highly allergenic in edible insects (98–101). Recently, the allergenic profile of two edible insect species, *A. domesticus* and *H. illucens*, was analyzed. Proteins from various commercial food products derived from these insects were examined, leading to the identification of allergens unique to each species, including hemocyanin, vitellogenin, HSP20, apolipophorin-III, and chitin-binding protein (102).

Although it has been reported that food proteins involved in allergic reactions may undergo alterations due to different processing methods (103), in particular, the allergenic properties of insect proteins remain largely unaffected by processes such as thermal processing and digestion (97). Previous research suggests that thermal processing methods such as blanching, baking, and frying are not entirely effective in eliminating the allergenicity in mealworms (104). However, other studies have reported that thermal processing in these insects leads to reduced IgE cross-reactivity to tropomyosin, suggesting a potential effect on the allergenic reactivity of these proteins (105). In the case of locusts (*Patanga succincta*), thermal processing revealed the persistence of various IgE-binding proteins in the sera of shrimp-allergic patients. While arginine kinase, enolase, and HEX1B were detected in raw locusts, fried locusts exhibited a reduction in these allergens, with only HEX1B and enolase remaining (106).

Notwithstanding these results are encouraging, they do not signify the end of efforts to eliminate allergens from insects and insect-based foods. Given the growing need and importance of incorporating insect-based foods into the food chain, further research on alternative processing technologies is crucial to evaluate their effectiveness in reducing allergic reactions to insect proteins and potentially eliminating their allergenicity.

## 6 Regulatory frameworks and guidelines for ensuring the safety of edible insects

As the global consumption of edible insects continues to rise, the need for regulatory frameworks to ensure consumer safety has become increasingly evident, leading several countries to implement specific regulations. In the European Union (EU), insects have been classified as “novel foods” since 2015, and their commercialization is regulated under Regulation (EU) 2015/2283 (107). The first insect received authorization for sale in 2021, and currently, four species have been approved: the yellow mealworm (*Tenebrio molitor*), the migratory locust (*Locusta migratoria*), the house cricket (*Acheta domesticus*), and the grain mold beetle larva (*Alphitobius diaperinus*). Their inclusion in human food products follows Regulation (EU) 2017/2470 (108), while specific implementing regulations govern the commercialization of *Acheta domesticus* (Regulation (EU) 2022/188) (109), *Tenebrio molitor* (Regulation (EU) 2022/169) (110), and *Alphitobius diaperinus* (Regulation (EU) 2023/58) (111). Since insects were not widely consumed before 1997, they are classified as novel foods and must be listed by the European Commission before they can be marketed, with labeling requirements specifying potential allergenic risks. In Canada, regulatory provisions allow food items with a documented history of traditional consumption elsewhere in the world to be sold without additional regulations, provided they undergo the novelty determination process (112). Consequently, various insect species have been classified as non-novel foods, and the Canadian government has officially recognized this ingredient as having a history of safe consumption (113). In the United States, edible insect products fall under the jurisdiction of the U.S. Food and Drug Administration (FDA) (114). In 2013, the FDA issued a “response to inquiry,” affirming that insects qualify as food under the Food, Drug, and Cosmetic Act (United States Code, Title 21) when intended for human consumption. This law stipulates that food must be clean, safe (free from contaminants, pathogens, and toxins), produced under sanitary conditions, properly stored and transported, and accurately labeled, as per Article 403 (114, 115). Furthermore, insect farming for human consumption must comply with current Good Manufacturing Practices (cGMP, 21CFR110), and insects bred for animal feed or collected from the wild cannot be marketed as food. If insect-based ingredients undergo modification or are used in food formulations, they may require authorization as food additives. Unless an insect-derived protein has GRAS (Generally Recognized as Safe) status, it is considered a food additive, though companies can independently determine GRAS status, with FDA notification being optional (115). In contrast, in Mexico, food safety regulations are overseen by two government agencies: The Ministry of Health and the Ministry of Agriculture, Livestock, Rural Development, Fisheries, and Food. However, there are currently no specific Official Mexican Standards regulating insect-based food products (114). Likewise, in Australia and New Zealand, there is no independent legal framework or specific government legislation for insect farming, prompting the Insect Proteins Association of Australia (IPAA) to establish guidelines for its members. In these countries, insect-based foods fall under Rule 1.5.1 of the Food Standards Code, which classifies them as novel foods requiring a safety assessment unless their safety has previously been established (114). Meanwhile, in China, any new raw material intended for food use, including high-protein insect ingredients, must receive approval from the Ministry of Health. Once added to the Food Materials Catalogue, the material becomes accessible to all food producers, though current food legislation does not explicitly mention insect-based products, with the exception of silkworm pupae, which were approved by the Ministry of Health in 2014 and included in the list of permitted food items (114). Finally, despite a long tradition of edible insect consumption, regulatory frameworks for edible insects in Africa remain highly fragmented, there is no specific legislation regulating insect farming or insect-based food products, and insects are not classified as novel foods in this region.

## 7 Conclusion

In conclusion, beyond their nutritional value, insects contain bioactive compounds with antioxidant, antimicrobial, immunomodulatory, and cardioprotective properties, making them potential functional foods with significant health benefits. Research indicates that insect-derived bioactive compounds may contribute to the prevention and management of chronic non-communicable diseases and oxidative stress-related conditions, further supporting their inclusion in human diets. However, several food safety concerns must be addressed to facilitate the broader acceptance and integration of edible insects into global food markets. Key challenges include the potential allergenicity of insect proteins, which share structural similarities with allergens found in crustaceans and dust mites, posing a risk to sensitive individuals. Additionally, microbial contamination, heavy metal accumulation, pesticide residues, and the presence of mycotoxins require strict monitoring to ensure consumer safety. Regulatory frameworks, such as those established by the European Food Safety Authority (EFSA), play a crucial role in setting safety standards and guiding best practices in insect farming, processing, and commercialization. As scientific research continues to explore the nutritional potential, bioactivity, and safety considerations of edible insects, they could become a key component of sustainable food systems, contributing to global nutrition, environmental sustainability, and human health. To fully unlock their potential, ongoing research, regulatory oversight, and technological advancements will be essential to ensure that edible insects are safe, nutritious, and widely accepted as a viable alternative food source.

## 8 Future perspectives

Considering the insights presented in this review, the future perspectives on edible insects as a nutritious, functional, and sustainable food source will largely depend on addressing key challenges related to food safety, consumer acceptance, regulatory frameworks, and technological advancements. Several areas require further exploration and development to maximize their potential and ensure their successful integration into global food systems.

1. Enhancing Food Safety and Allergen Management

- Development of improved processing technologies: Future research should focus on identifying and optimizing processing techniques that can reduce allergenicity while maintaining the nutritional and functional properties of edible insects. Alternative methods such as fermentation, enzymatic hydrolysis, or advanced thermal treatments should be explored.
- Risk assessment of contaminants: Continuous monitoring of heavy metals, pesticides, mycotoxins, and microbial pathogens in insect farming is crucial. Further studies should establish safe thresholds for contaminants and develop effective detoxification strategies.
- Characterization and labelling of allergens: More comprehensive research is needed to identify and characterize insect allergens and assess cross-reactivity with common food allergens (e.g., shellfish). Implementing clear allergen labelling regulations and consumer education initiatives will be essential.

1. Expanding Research on Functional and Health Benefits

- Clinical trials on bioactive compounds: While *in vitro* and animal studies suggest antioxidant, antimicrobial, immunomodulatory, and cardioprotective properties, human clinical trials are needed to confirm the health benefits of insect-derived bioactive compounds.
- Investigating the gut microbiome effects: Research on how insect consumption influences gut microbiota composition and metabolic health should be expanded, especially considering the prebiotic potential of chitin and insect-derived fibers.
- Personalized nutrition and insects: Future studies should assess how individual dietary needs, genetics, and gut microbiota profiles influence the metabolism and bioavailability of insect-based nutrients.

1. Strengthening Regulatory Frameworks and Standardization

- Harmonization of global regulations: Currently, regulatory standards for edible insects vary across regions (e.g., EFSA in Europe, FDA in the USA). A globally unified approach to safety, labelling, and processing standards will facilitate market expansion and consumer trust.
- Establishing quality control protocols: Guidelines should be developed for insect farming, harvesting, and processing, ensuring traceability, hygiene, and sustainability in production chains.
- Integration into national food policies: Governments and international organizations should incorporate edible insects into nutritional programs and food security policies, especially in regions where malnutrition remains a challenge.
- Policy recommendations for sustainable production: Future policies should focus on incentivizing sustainable farming practices, reducing environmental impact, and ensuring ethical considerations in large-scale insect farming operations.

1. Consumer Acceptance and Market Expansion

- Overcoming psychological and cultural barriers: While edible insects are traditionally consumed in many regions, Western markets still show reluctance. Educational campaigns, culinary innovations, and attractive product formulations (e.g., protein bars, insect-enriched flours, or snacks) will be key to increasing consumer acceptance.
- Mainstreaming insect-based products: Collaboration between the food industry, chefs, and researchers can help incorporate edible insects into widely accepted food products, making them more palatable and accessible to different demographics.
- Sustainability labelling and marketing: Highlighting the environmental benefits of edible insects (e.g., low carbon footprint, reduced water use, and high feed conversion efficiency) can appeal to eco-conscious consumers.
- Policy initiatives to promote insect-based foods: Governments should consider introducing incentives or subsidies to encourage the adoption of insect-based ingredients in food formulations, particularly as a sustainable alternative to traditional livestock-based proteins.

1. Technological Innovations in Insect Farming and Processing

- Automation and scalability: Advancements in precision farming, AI-driven monitoring systems, and automated rearing facilities will be essential for scaling up insect farming while maintaining efficiency and quality control.
- Nutrient optimization through feed formulation: Future research should explore how different feeding substrates affect the nutritional composition of edible insects, optimizing their protein, fatty acid, and bioactive compound content.
- Biotechnological applications: Genetic engineering and fermentation technologies could be leveraged to enhance the nutritional profile, digestibility, and functionality of insect-derived ingredients.
- Developing alternative protein extraction methods: Research should focus on improving protein isolation techniques from edible insects, ensuring high purity, stability, and bioavailability in food applications.
